# Vaccination in Turkey

**Published:** 1828-01

**Authors:** 


### VACCINATION.

#### *Vaccination in Turkey.*

Sir,—I hope you will find it convenient to insert in your next Number the accompanying interesting letter. It is translated from a copy in French, which has been transmitted to me within these few days, by Dr. De Carro. The facts which it discloses, whether considered morally, medically, or politically, are very curious. He intended that it should appear in the second volume of my Life of Jenner; but as some time must elapse before it can be published, I think it wrong to withhold an account of this signal triumph of vaccination over national and religious prejudices till that event takes place. I therefore transmit Dr. A.'s letter for your Journal, which, during a long series of years, has evinced unwearied zeal and diligence in recording every important circumstance connected with the vaccine discovery.

I have the honour to remain, sir, most faithfully yours,

J. BARON.

*Gloucester; November* 2, 1827.

*Copy of a Letter from* Dr. Auban, *a French Physician, settled at Constantinople for upwards of thirty years, to* Dr. De Carro, *formerly of Vienna, now of Prague.*

If during so long a time I have not given you any sign of my being alive, it is because vaccination in this country no longer offered any thing interesting: but an event, which no one could have surmised, and which, in consequence, has astonished all those who have been made acquainted with it, ought to be transmitted to you.

Before announcing it to you, I should remind you that no Christian is ever permitted even to touch any prince of the Ottoman race, or still less to take blood from him in any way, or on any account. The great revolution that has been effected among the Massulman people since the destruction of the Janissaries has changed every thing! The troops placed on the footing of other European soldiers,—the musket with its bayonet,—the military music, and nothing played except European airs,—a drum-major, with his great cane in his hand,—the sappers preceding the regiments,—the Grand Seigneur himself in general's uniform, ordering certain manœuvres,—all these are the prodigies which one can with difficulty comprehend, and the whole brought about in a very short time indeed by one individual, but he, in truth, a great man!

Vaccination performed the 16th of May on three Sultans, or Sultanes, (a title given only to infants who are born on the imperial throne,) and two other young ladies of the harem, proves how much every thing has been changed amongst this people.

On the 14th of May, one of the physicians of the Sultan's seraglio begged of me to go to his apartments. He told me that he had received a message from the Echim Bachi, directing him to request of me to hold myself in readiness to go to vaccinate the children of the Sultan,—to have the vaccine matter always about me, and not to remove any distance from Pera. I remarked to him, that intrigues would cause some other person to be chosen to perform that operation. He replied to me, "There are no longer any intrigues that can cause the order of the Sultan himself to be altered, who has pointed out you on account of your age, your nation, and your name."

On the 16th, in the morning, an order for me to go to the palace with one of the physicians, who would act as interpreter, was transmitted to me. About nine o'clock we were shown into a chamber allotted to the Echim Bachi, who made no delay in coming. He caused the Kislar Aga (the chief of the black eunuchs) to be sent for, and immediately we three were introduced. At the first chamber where we stopped, we found a young sultan, seven or eight months old, who was vaccinated forthwith. A few minutes afterwards his elder sister, about a year and a half old, appeared; she was also vaccinated; and then was brought in a still younger princess, who was submitted to the same operation: and all this took place without the smallest difficulty or ceremony. In two other apartments, two young ladies were also vaccinated.

The verification was adjourned to the 23d of the same month. The Echim Bachi, being sick, did not come; but we were introduced notwithstanding, and all the persons vaccinated were found going on well, with a most beautiful (*tres-belle*) vaccine pock.

The 28th of the same month we returned to the palace, and the crusts which had formed left no fear about the maturation of the vesicles: all was finished, and complete. The Kislar Aga remitted me a very handsome present on the part of his highness, adding, "I have received this from the hand of the Grand Seigneur, to be given into yours. He has sent it to you also to testify his satisfaction with you. That which you received the first day was sent to you on the part of the mother of the two young princes. We will now go home, to return no more to this place until some new prince be born."

You have here, as I think, a piece of news which ought to be transmitted to you. If you wish for farther details, let me know, and I shall consider it a duty to satisfy you. All this was done without the least mystery.

